# Impact of a High-Fat High-Carbohydrate (HFHC) Diet at a Young Age on Steroid Hormone Hair Concentrations in Mice: A Comparison with a Control Diet and Nutraceutical Supplementation

**DOI:** 10.3390/life15111722

**Published:** 2025-11-07

**Authors:** Isabella Pividori, Tanja Peric, Antonella Comin, Natalia Rosso, Silvia Gazzin, Mirco Corazzin, Alberto Prandi

**Affiliations:** 1 Department of Agricultural, Food, Environmental and Animal Sciences, University of Udine, 33100 Udine, Italy; isabella.pividori@uniud.it (I.P.); antonella.comin@uniud.it (A.C.); mirco.corazzin@uniud.it (M.C.); alberto.prandi@uniud.it (A.P.); 2 Fondazione Italiana Fegato ONLUS-Italian Liver Foundation NPO, AREA Science Park Basovizza, 34149 Trieste, Italy; natalia.rosso@fegato.it (N.R.); silvia.gazzin@fegato.it (S.G.)

**Keywords:** mouse, cortisol, DHEA, DHEA-S, progesterone, 17-β-oestradiol, testosterone, silymarin, coconut oil

## Abstract

An unhealthy prepubertal diet can have long-lasting effects throughout life. This study investigated hair concentrations of adrenal and sex steroids, in an in vivo mouse model of juvenile obesity subjected to control (CTRL), obesogenic (HFHC) diet, or nutraceutical supplementation (silymarin or coconut oil) diets. 87 3-week-old C57BL/6 mice (42 females, 45 males) were fed CTRL or HFHC diets for 8 weeks. Afterward, the CTRL group continued on CTRL diet while the HFHC diet group was divided into five groups: HFHC, HFHC→CTRL, HFHC→CTRL + silymarin (SIL), HFHC→HFHC + SIL and HFHC→HFHC + Coconut oil. At 4 weeks, the HFHC group showed increased cortisol/dehydroepiandrosterone (DHEA) ratio compared to CTRL group. At 20 weeks, the HFHC→HFHC group showed higher levels of progesterone (P4) and dehydroepiandrosterone sulfate (DHEA-S) and lower levels of estradiol (E2) compared to the CTRL→CTRL group. The switch from HFHC→CTRL was the optimal therapy because the body weight and almost all the hormones were close to those observed for the CTRL diet group. Supplement with SIL or Coconut oil reduced DHEA-S and increased in E2 compared with the endocrine setting seen with the HFHC diet. These interventions should be considered as supportive measures rather than substitutes for dietary correction.

## 1. Introduction

The common forms of obesity often have their roots in childhood. An unhealthy diet during the prepubertal period can be particularly dangerous, with effects that may extend over the course of a lifetime. Overweight and obese children are likely to remain obese into adulthood and are more likely to develop noncommunicable diseases [1], such as heart disease, diabetes, insulin resistance, liver steatosis, Cushing’s syndrome and depression [2,3,4]. Furthermore, food overload over long periods leads to a breakdown in the regulatory mechanisms of the hypothalamic–pituitary–adrenal (HPA) and hypothalamic–pituitary–gonadal (HPG) axes, with consequent endocrine imbalances and reproductive disorders [5,6].

Obesity is a complex condition associated with changes in many steroid hormones [7] that can affect the process of growth and puberty [8]. Excess adiposity may influence various aspects of pubertal development, such as the timing of pubertal initiation and the hormonal concentrations of sex steroids during puberty [9], and it may promote the development of adolescent polycystic ovary syndrome [10] as well as the onset of breast cancer [11]. The literature suggests that, although there are still conflicting reports, compared with normal-weight children, overweight children have higher cortisol [12], DHEA (S), testosterone [13], progesterone (P4) [14] and 17-β-estradiol (E2) [15] snapshot concentrations.

Closely associated with obesity, metabolic dysfunction-associated steatotic liver disease (MASLD) (formerly called nonalcoholic fatty liver disease (NAFLD) and nonalcoholic steatohepatitis (NASH)) has become the most common cause of chronic liver diseases in obese children [16]. While MASLD (characterized by simple steatosis) is a reversible (by lifestyle changes) and benign disorder, the progression toward the more severe metabolic dysfunction-associated steatohepatitis (MASH) must not be disregarded [17]. Diet, particularly a hypercaloric diet, plays a role in the pathophysiology of MASLD and MASH [18,19,20]. A study conducted in a juvenile (C57BL/6J) mouse model of diet-induced obesity reproducing MASH, revealed that a high-fat high-carbohydrate diet (HFHC), a significant increase in body weight, BMI, visceral adiposity, dyslipidemia, hyperglycemia, hyperinsulinemia, and some degree of hepatic fibrosis both in males and females [17,21].

Currently, treatment options are limited; however, there is broad consensus that dietary intervention remains the cornerstone of management. Among the most effective dietary models, the Mediterranean diet has been shown to improve metabolic and hepatic profile [22]. To date, first-line therapy for treating obesity and its associated pathologies is based on lifestyle changes to induce weight loss through caloric restriction and physical activity [23]. Recent studies [24,25] have explored the use of bioactive food compounds to counteract obesity, identifying this as an attractive therapeutic approach.

Antioxidant, anti-inflammatory, and hepatoprotective properties of some substances naturally present in foods and plants, such as silymarin and coconut oil, suggest their feasible role in assisting with many pathophysiological conditions. In particular, silymarin, a flavonoid extracted from plant seeds and fruits of *Silybum marianum* [26], has antioxidant activity with hepatoprotective, anti-inflammatory and anticholesterolemic properties [27,28] and has been widely proposed for the treatment of MASH. In an experimental mouse model of diet-induced obesity that reproduce many of the features involved in the onset of MASLD, investigated hepatic and metabolic changes and shows that orally administered silymarin, which is incorporated into the Western diet, can be a strategy to prevent/revert damage related to the HFHC diet [17].

Several studies have also demonstrated the efficacy of coconut oil among hepatoprotective plants. In addition, coconut oil has attracted attention because of its hypocholesterolemic, anticancer, antiobesity, antihepatosteatotic, antioxidant, anti-inflammatory and cardioprotective effects [29]. Among these functions, antioxidants are the most basic and important and are determined mainly by phenolic compounds and medium-chain fatty acids [30]. In the literature, different biologically active substances and their effects on organisms have been tested in blood, oral liquids (saliva), urine, feces, tissues, nails or claws and hair [31,32]. Unlike traditional matrices (serum, saliva, urine, or feces) that are subjected to circadian fluctuations and provide a measurement of the concentration at a single time point or within a 12–24 h period when testing for hormonal concentrations, hair analysis is unaffected by these variations or by factors that induce short-term variations. Furthermore, hair provides a retrospective calendar of exposure to hormones and an integrated measure of hormone concentrations over medium- and long-term periods in mice [33] and in rats [34,35]. Moreover, hair growth cyclical analysis can reflect different physiological states. The hair growth cycle consists of three distinct phases: anagen (growth phase), catagen (regression phase) and telogen (resting or quiescent phase). In mice, this process is neither entirely asynchronous nor completely synchronized; instead, hair growth occurs in waves that dynamically propagate across the skin. As a result, neighboring areas of the skin can simultaneously contain hair follicles at different stages of the cycle [36]. Hair provides information for objectively evaluating long-term exposures, sampling is not invasive or painful, and samples can be easily stored at room temperature for an extended period [37]. This matrix might be a valuable tool for studying the interaction between a diet and steroidogenesis over long periods of time [4,38,39,40,41].

The aim of the present study was to investigate the hair concentrations of adrenal and sex steroids in an in vivo mouse model of juvenile obesity subjected to a CTRL diet, an obesogenic (HFHC) diet, and nutraceutical supplementation (silymarin or coconut oil).

## 2. Materials and Methods

### 2.1. Ethical Approval

Animal care and procedures were conducted in accordance with Italian and European law and approved by the National Authority (Ministero della Salute, Italy, Approval N° 11072013). Regular communication with the local authority was performed. The maximal effort was made to reduce the number of animals used and their suffering with respect to the 3R rule.

### 2.2. Experimental Setup

#### 2.2.1. Animal Model

The 87 C57Bl/6 mouse pups (42 females and 45 males) were obtained from Harlan Laboratories S.R.L. (San Pietro al Natisone, Italy). To minimize the stress related to handling, after weaning (3 weeks of age), the pups were handled by a single expert operator and immediately and randomly housed in cages assigned to the experimental protocol in a temperature-controlled environment (22 ± 2 °C) with a 12 h light/dark schedule.

Body weights were recorded at the baseline (3 weeks of age) and immediately prior to sacrificed to determine final live weight (LW). Animals were deeply anesthetized with Zoletil (10 mg/kg) and Xylazine (5 mg/kg) via intraperitoneal injection.

The experimental model spans the developmental period from weaning to adulthood. In humans, this corresponds to the age range of 3 to 30 years, a period during which the incidence of obesity has reached epidemic proportions [17].

#### 2.2.2. Animal Feeding

**Figure 1:**
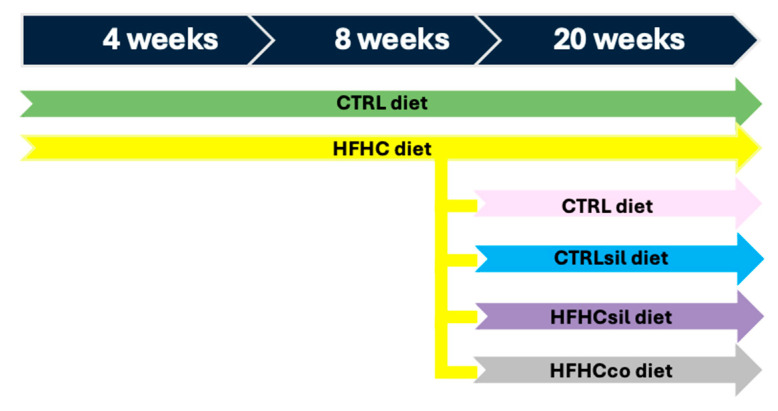
In vivo experimental setup. The experiments started at 3 weeks of age (after weaning), and the animals were divided into two groups, one fed the CTRL (green arrow) diet and the other fed the high-fat high-carbohydrate (HFHC) (yellow arrow) diet, for a total of 8 weeks. After 8 weeks of treatment, the CTRL group and part of the HFHC group were fed the original diets and served as controls (CTRL→CTRL; green arrow; HFHC→HFHC; yellow arrow), whereas the rest of the HFHC group was divided into four groups. These groups were switched to the CTRL diet (HFHC→CTRL; pink arrow), to the CTRL diet enriched with silymarin (silymarin-formulated CTRL; HFHC→CTRLsil; blue arrow), to the HFHC diet enriched with silymarin (silymarin-formulated HFHC; HFHC→HFHCsil; violet arrow) and to the HFHC diet enriched with coconut oil (coconut oil-formulated HFHC; HFHC→HFHCco) (gray arrow).

After weaning (at 3 weeks of age), the animals were divided into two groups (see Figure 1): mice were fed ad libitum with a control diet (CTRL, D12328, Research Diets, New Brunswick, NJ, USA; 16% kcal protein, 73% kcal carbohydrate, 11% kcal fat in the form of soybean oil and hydrogenated coconut oil) or a HFHC diet (HFHC, D12331, Research Diets, New Brunswick, NJ, USA; 16.4% kcal protein, 25.5% kcal carbohydrate, 58% kcal fat in the form of soybean oil and hydrogenated coconut oil, respectively) plus 42 g/L fructose/sucrose (55% fructose/45% sucrose) in the drinking water for 8 weeks (24 mice, 12 male and 12 female assigned in a balanced manner to the experimental groups), which is the time required for the onset of MASH, as described previously by Marin et al. (2016) [21]. After 8 weeks, the HFHC diet group was divided into four groups with different diets. The first group of animals was switched to a CTRL diet (HFHC→CTRL; 9 mice; 5 male, 4 female), the second group was switched to a CTRL enriched with silymarin (HFHC→CTRLsil; 7 mice; 3 male, 4 female), the third group was fed a HFHC enriched with silymarin (HFHC→HFHCsil; 14 mice; 8 male, 6 female), and the fourth group was fed a HFHC enriched with coconut oil (HFHC→HFHCco; 11 mice; 6 male, 5 female). Some of the animals continued the HFHC (10 mice; 5 male, 5 female) and CTRL diets (12 mice; 6 male, 6 female) and were used as references. The HFHC diet was changed after 8 weeks of treatment when the mice exhibited marked weight gain, dyslipidemia and moderate hepatic steatosis [21]. It is considered the proper time to act in the moment in which the disease is still in a reversible stage [17], given that Marin et al. (2016) [21] reported that eight-week-old animals presented with histological signs of MASH.

#### 2.2.3. Nutraceutically Supplemented Diets

As previously described by Marin et al. (2017) [17], for physiological administration, silymarin extract (Sigma Aldrich, St. Luois, MO, USA) containing 210 mg/g silybin was included both the CTRL (CTRLsil) and the HFHC (HFHCsil) diets. Briefly, the composition of the animal food provided by Research Diets Inc. (Research Diets, New Brunswick, NJ, USA) and designed for a CTRL or an HFHC diet was modified opportunely to include silymarin in the product (D12328, 16% kcal protein, 73% kcal carbohydrate, 11% kcal fat in the form of soybean oil and hydrogenated coconut oil; or D12331, 16.4% kcal protein, 25.5% kcal carbohydrate, 58% kcal fat in the form of soybean oil and hydrogenated coconut oil, respectively). In particular, silymarin powder (0.01 g/g) and Tween 80 (Sigma Aldrich, St. Luois, MO, USA) (0.1 g/g) were added to coconut oil (Unigrà, Conselice, Italy) previously heated at 40 °C. The system was maintained at 40 °C until silymarin solubilization was complete. The oil enriched with silymarin was manually incorporated at a concentration of 14% by weight in paste obtained by grounding the animal feed pellet with a mixer (Kenwood, Prospero KM260, Havant, UK). The silymarin-enriched paste was subsequently subdivided into aliquots of 4.5 g cylinders. The effective concentration of the bioactive component (silybin) contained in each food cylinder was 270 mg/kg, as determined by HPLC analysis [17]. Similarly, the HFHCco diet included coconut oil (Unigrà, Conselice, Italy) at the same concentration as the silymarin described above for the HFHCsil diet.

### 2.3. Hair Sampling

All hair samples were collected prior to sacrifice. Hair samples were carefully and painlessly taken from the backs of the animals via an electric razor. Each sample was immediately stored in a paper envelope, at room temperature in a dry room until it was processed. At each experimental check-point, a single sample of hair was collected at different physiological phases (anagen, catagen, and telogen). Consequently, the concentrations found in mouse hair represent the integral of blood concentrations of a time frame starting from −21/−15 days of sampling because of its initial deposition in the underlying skin [42]. Considering this lag time and considering that mice reach sexual maturity at 8–12 weeks of age [43], hair samples collected at 4 and 8 weeks of treatment (7–11 weeks of life) were from sexually immature animals. Hair samples collected at 20 weeks of treatment refer to adult animals that have reached sexual maturity. Based on previous data [44,45], the complete hair growth cycle in C57BL/6J mice lasts approximately 3 weeks.

The hair samples were stored in a dry envelope at room temperature in the dark until analysis. To avoid stress and remain in compliance with current legislation on animal welfare, samples were collected during routine procedures in animal facility.

### 2.4. Hair Sample Preparation

Sixty milligrams of hair were weighed, and each hair strand was washed twice with 3 mL of isopropanol (≥99.8%, Sigma-Aldrich) for 3 min. Steroids were extracted, in agreement with Davenport et al. (2006) [46], by incubating each sample for 16 hours in methanol (≥99.8%, Avantor Chemicals, Radnor, PA, USA) at 37 °C. Next, the liquid in the vial was evaporated to dryness at 37 °C under an airstream suction hood. The dried residue was then resuspended in 0.4 mL of 0.05 M phosphate-buffered saline (PBS), pH 7.5 (RIA buffer).

### 2.5. Hormones Assays

The concentrations of cortisol, DHEA, DHEA-S, P4, E2 and testosterone were measured using a solid-phase microtiter RIA. In brief, a 96-well microtiter plate (OptiPlate; PerkinElmer Life Sciences, Boston, MA, USA) was coated with goat anti-rabbit γ-globulin serum diluted 1:1000 in 0.15 mM sodium acetate buffer (pH 9) and incubated overnight at 4 °C. The plate was then washed twice with RIA buffer (pH 7.5) and incubated overnight at 4 °C with 200 μL of the antibody serum (Table 1).

After washing the plate with RIA buffer, the standards (5–200 pg/well), the quality-control extract, the test extracts, and the tracer (hydrocortisone {cortisol [1,2,6,7-^3^H (N)]-}, DHEA [1,2,6,7-^3^H (N)], DHEA-S [1,2,6,7-^3^H (N)], progesterone [1,2,6,7-^3^H (N)], 17-β-oestradiol [2,4,6,7-16-17-^3^H (N)] and testosterone [1,2,6,7-^3^H (N)]) were added, and the plate was incubated overnight at 4 °C. The bound hormone was separated from the free hormone by decanting and washing the wells in RIA buffer. After the addition of 200 μL of scintillation cocktail, the plate was counted on a β-counter (Perkin-Elmer Life Science, Boston, MA, USA).

### 2.6. Statistical Analysis

Statistical analysis was performed using SPSS for Windows, version 29.0 (SPSS Inc Chicago, IL, USA), and R software, version 4.1.2. The normality of the data distribution was tested using the Shapiro–Wilk test. When appropriate, the data were transformed for parametric testing. Within weeks of treatment (at 4, 8 and 20 weeks), the effects of the diet on LW and hair hormone concentrations and their ratios were analyzed via a general linear model that considers diet as fixed factor and sex as fixed control factor to account for possible sex-related variability.

y_ijk_ = μ + Diet_i_ + Sex_j_ + ε_ijk_

The normality of the data distribution was tested using the Shapiro–Wilk test. When appropriate, the data were transformed and the same model was used. Multiple comparisons between means were performed via Sidak test. Significance was considered at *p* ≤ 0.05, and a trend towards significance was considered at *p* ≤ 0.10. In text data are reported as estimated marginal mean, 95% confidence interval: lower-upper–upper.

## 3. Results

C57BL/6 mice were exposed to a HFHC diet immediately after weaning (3 weeks old). Eight-week-old HFHC (obesogenic) diet-fed animals presented marked weight gain and histological signs of MASH compared with C57BL/6 mice receiving a control diet (CTRL) [19]. Table 2 shows the effects of the HFHC diet on LW, hormones and their ratios in the hair of mice after 4 weeks of treatment. The HFHC group exhibited a significant LWs than the CTRL group (*p* < 0.01). A HFHC diet interfered with the HPA axis in mice, resulting in a significant increase in the cortisol/DHEA ratio (*p* < 0.01) due to a decrease in DHEA (*p* = 0.02) and a tendency toward an increase in the hair cortisol concentration (*p* = 0.06). DHEA-S concentrations remained unchanged (47.60, 37.21–58.00, pg/mg; *p* = 0.20), as did the cortisol/DHEA-S*100 ratio (3.16, 2.74–3.58; *p* = 0.49), whereas the DHEA/DHEA-S ratio was significantly reduced (*p* < 0.01) after 4 weeks of a HFHC diet. Additionally, the HFHC diet tended to affect sex hormones in mice. Compared with the CTRL diet, the HFHC diet tended to increase testosterone (*p* = 0.09) and significantly increased the concentrations of both P4 (*p* = 0.04) and E2 (*p* = 0.04) without affecting the testosterone/E2 (5.03, 4.06–5.99; *p* = 0.17) and cortisol/testosterone ratios (0.25, 0.19–0.32; *p* = 0.66). Male mice showed greater live weight (24.8, 24.0–25.5, vs. 19.1, 18.3–19.9, g; *p* < 0.01), testosterone (9.12, 6.38–11.86, vs. 4.71, 1.97–7.45, pg/mg; *p* = 0.03), testosterone/E2 ratio (6.73, 5.36–8.10, vs. 3.32, 1.95–4.69, pg/mg; *p* < 0.01), but lower DHEA/DHEA-S ratio (1.38, 1.20–1.57, vs. 1.80, 1.62–1.98; *p* < 0.01) and cortisol/testosterone ratio (0.17, 0.07–0.26, vs. 0.34, 0.25–0.43; *p* = 0.01) compared to female mice. Conversely, sex did not influence cortisol (*p* = 0.97), DHEA (*p* = 0.35), cortisol/DHEA*100 (*p* = 0.60), P4 (*p* = 0.43), E2 (*p* = 0.27) and DHEAS (*p* = 0.20).

The effect of the HFHC diet in mice after 8 weeks was similar but less pronounced than that observed after 4 weeks (Table 3). In particular, the HFHC-fed mice tended to have a greater LW than the CTRL-fed mice did (*p* = 0.09). Considering the HPA axis, the HFHC group tended to have higher cortisol (*p* = 0.09) but a similar DHEA concentration (63.80, 58.60–68.99, pg/mg; *p* = 0.24) than the CTRL group did, leading to a tendency toward a higher cortisol/DHEA ratio (*p* = 0.06). Furthermore, the HFHC diet reduced the DHEA/DHEA-S ratio (*p* = 0.01) without affecting the DHEA-S concentration (44.75, 39.94–49.56, pg/mg; *p* = 0.24) or both the cortisol/testosterone ratio concentration (0.30, 0.23–0.36; *p* = 0.29) and the cortisol/DHEA-S *100 ratio (3.60, 3.10–4.10; *p* = 0.66). Compared with the CTRL diet, the HFHC diet significantly increased the concentrations of both P4 (*p* = 0.02) and E2 (*p* = 0.05). The concentrations of testosterone (5.95, 5.02–6.88, pg/mg; *p* = 0.85) were not affected by diet. Male mice showed greater testosterone (7.98, 6.67–9.29, vs. 3.93, 2.61–5.24, pg/mg; *p* < 0.01), testosterone/E2 ratio (6.12, 4.79–7.45, vs. 2.91, 1.71–4.12; *p* < 0.01), DHEA-S (55.06, 48.26–61.86, vs. 34.44, 27.64–41.24, pg/mg; *p* < 0.01), but lower DHEA/DHEA-S ratio (1.27, 1.11–1.43, vs. 1.73, 1.57–1.89, *p* < 0.01) and cortisol/testosterone ratio (0.22, 0.12–0.31, vs. 0.38, 0.28–0.47, *p* = 0.02) compared to female mice. Conversely, sex did not influence live weight (*p* = 0.58), cortisol (*p* = 0.15), DHEA (*p* = 0.07), cortisol/DHEA*100 ratio (*p* = 0.63), P4 (*p* = 0.93), cortisol/DHEA-S*100 ratio (*p* = 0.051) and E2 (*p* = 0.89).

Table 4 shows the effects of diet type on LW, hormones and their ratios in the hair of mice after 20 weeks. The LW was lowest in the mice receiving the CTRL (*p* < 0.01; CTRL→CTRL, HFHC→CTRL, HFHC→CTRLsil). Cortisol concentrations were greater in the HFHC→HFHCsil group than in the HFHC→HFHCco and CTRL→CTRL groups (*p* ≤ 0.05). However, because the DHEA concentration was similar between the experimental groups (69.97, 73.43–66.82, pg/mg; *p* = 0.91), an effect of diet type on the cortisol/DHEA*100 ratio was not detected (2.69, 2.52–2.88, pg/mg; *p* = 0.11). The DHEA-S concentration was greater in the HFHC→HFHC group than in the CTRL→CTRL, HFHC→CTRL and HFHC→HFHCco groups (*p* ≤ 0.05). In contrast, the cortisol/DHEA-S ratio was lower in the HFHC→HFHC group than in the CTRL→CTRL, HFHC→CTRL and HFHC→HFHCsil groups (*p* ≤ 0.05). Considering sex hormones, testosterone concentrations (12.98, 11.24–14.99, pg/mg; *p* = 0.41) and the cortisol/testosterone ratio (0.12, 0.11–0.14; *p* = 0.11) were not influenced by the type of diet (*p* > 0.05). Conversely, E2 was highest in the CTRL→CTRL group and lowest in the HFHC→HFHC group (*p* ≤ 0.05), with the other experimental groups showing intermediate values. The opposite trend was observed for the testosterone/E2 ratio. P4 concentrations were greater in the HFHC→HFHC and HFHC→HFHCsil groups than in the CTRL→CTRL and HFHC→CTRL groups (*p* ≤ 0.05). Male mice showed greater live weight (37.64, 36.23–39.04, vs. 28.86, 27.41–30.31, g; *p* < 0.01), testosterone/E2 (3.34, 2.67–4.14, vs. 1.91, 1.45–2.46, *p* < 0.01), DHEA-S (62.89, 57.43–68.85, vs. 43.39, 39.51–47.65; *p* < 0.01), testostosterone (15.20, 12.11–19.09 vs. 11.08, 8.76–14.02 pg/mg; *p* = 0.03) but lower cortisol (1.73, 1.56–1.89, vs. 2.20, 2.04–2.37, pg/mg; *p* < 0.01), P4 (22.12, 19.20–25.04, vs. 35.30, 32.28–38.32, pg/mg; *p* < 0.01), E2 (5.16, 4.13–6.45, vs. 7.14, 5.68–8.99, pg/mg; *p* = 0.02), DHEA/DHEA-S ratio (1.18, 1.07–1.29, vs. 1.66, 1.55–1.78; *p* < 0.01), cortisol/DHEA-S*100 ratio (2.77, 2.41–3.14, vs. 5.12, 4.75–5.50; *p* < 0.01), cortisol/DHEA (2.35, 2.11–2.61 vs. 3.08, 2.76–3.44; *p* < 0.01), cortisol/testosterone ratio (0.10, 0.08–0.12 vs. 0.17, 0.13–0.25; *p* < 0.01) compared to female mice. Conversely, sex did not influence DHEA (*p* = 0.54).

## 4. Discussion

Obesity, which results from excess body mass, seems to be linked to multiple dysregulations in steroid synthesis, metabolism, and excretion [47,48].

Considering that obesity often starts at an early age in humans and that information on steroid hormones in obese children is limited and controversial [14,47], our goal was to evaluate the effects of an obesogenic diet (HFHC) and corrective diets (HFHC→CTRL; HFHC→CTRLsil; HFHCsil; HFHCco) on variations in hair of some steroid hormones of the HPA and HPG axes in an in vivo mouse model of juvenile obesity. These hormones are involved in the maintenance of homeostasis [49] and in the metabolism, accumulation and distribution of adipose tissues [50].

The survey conducted on the hair of an obese mouse model was useful for investigating the long-term effects of an obesogenic diet on the concentrations of steroid hormones since hair incorporates systemic steroids into the growing hair shaft from blood vessels via passive diffusion during its growing phase—anagen [51]. The administration of a HFHC diet for an extended period of time can be considered a stressor that induces obesity, loss of homeostasis and stress. The HPA axis is activated to help animals cope with stress and regain physiological balance.

At 4 and 8 weeks of treatment, prepubertal mice fed the HFHC diet presented HPA axis stimulation, which was more evident at 4 weeks of treatment. Until the end of the period in which the dysfunction induced by the administration of the HFHC diet was still in a reversible stage [47], we observed, as in a previous study on hair [52], a significantly increased cortisol/DHEA ratio due to the tendency of cortisol to increase and a significant reduction in DHEA concentrations. These findings suggest that an initial physiological imbalance and loss of resilience are present in HFHC-treated animals. Disruption of the dynamic balance of these two hormones, especially a relatively high cortisol/DHEA(S) ratio, has an impact on psychological and physical health [53,54,55]. Moreover, the reduction in hair concentrations of DHEA observed in prepubertal animals was in agreement with other authors who reported a significant negative correlation between DHEA concentrations in human plasma and the development of metabolic diseases such as obesity, diabetes, cardiovascular disease, and hyperlipidemia [56]. Furthermore, the literature has shown that DHEA in rodents reduces body weight and visceral fat and increases glucose and insulin concentrations, which produces an antiobesity effect [57,58]

At 20 weeks of treatment, the cortisol/DHEA ratio in pubertal mice that continued to be fed the HFHC diet was adapted to the obesogenic diet compared with that in mice that continued to be fed the CTRL diet. Moreover, significantly greater DHEA-S concentrations were observed in the HFHC→HFHC group than in the CTRL→CTRL group, and this increase was mirrored by a significant reduction in the DHEA/DHEA-S ratio at 4 and 8 weeks of treatment, highlighting the effect of the diet on steroids. As described by Vale et al. (2015) [59], both DHEA and DHEA-S play common roles, but several differences exist in their mechanisms of action. To date, contradictory results have been reported in terms of the relationships between DHEA-S and adiposity [56]. Some studies on human plasma have failed to show an association between adiposity and DHEA-S [60]), whereas others have shown an association [61,62,63,64], as we observed in our study. The precise physiological role, mechanism(s) and target tissues of this hormone are still controversial [65]. In addition to its conversion to estrogens and androgens, DHEA-S has positive effects on a heterogeneous set of diseases and obesity-related pathologies, e.g., improving glucose metabolism in type 2 diabetic patients, reducing the risk of cardiovascular disease, decreasing fat mass and even preventing adipocyte differentiation and proliferation with a better metabolic profile toward a modification of the adipose tissue fatty acid profile [65].

The sex hormones of prepubertal mice subjected to the HFHC diet compared with those of mice fed the CTRL diet tended to increase, while significantly greater concentrations of both E2 and P4 were observed at 4 weeks of treatment. High concentrations of these hormones have also been reported by other authors in human [14,66] and mouse [67] plasma from obese individuals. In agreement with these results, Cao et al. (2019) [68] reported high serum concentrations of P4 in prepuberal overweight children.

Our results still revealed that at 20 weeks of treatment, in pubertal mice, significantly higher P4 concentrations were detected in the mice fed the obesogenic diet than in the CTRL animals. Given that P4 is a precursor of glucocorticoids, testosterone, and estrogens, one possible explanation for the increase in P4 could involve a heightened requirement for steroid precursors [67]. The literature on P4 and obesity is contradictory, and it would be more rigorous to acknowledge the lack of consensus and to explore alternative explanations (e.g., adrenal hyperactivity).

Regarding E2 hair concentrations were consistent with those obtained by Vandewalle et al. (2015) [69] but contrasted with the findings of Whyte et al. (2007) [70], particularly in relations to human plasma and mouse serum. Notably, the hair concentrations of E2 in adult mice fed the HFHC diet were significantly lower than those in CTRL animals, indicating a diet-induced impairment of estrogenic homeostasis in the HFHC group. In the literature, excessive fat accumulation is characterized by altered expression of estrogen receptors and key enzymes involved in their synthesis [71]. Importantly, given the inhibitory influence of estrogens on adipogenesis and lipogenesis its deficiency leads to excessive fat accumulation and impairs adipocyte function [71]. Jones et al. (2007) [72] reported that the aromatase-knockout mouse (ArKO), a model of estrogen deficiency, exhibited excessive adiposity and apoptosis of dopaminergic neurons in the hypothalamic arcuate nucleus, an area critical for regulation energy intake, storage, and mobilization, underscoring the significance of the testosterone/E2 ratio. In our study, this ratio was significantly greater in adult HFHC diet-fed mice than in CTRL-fed mice, which is in line with the observations of Jones et al. (2007) [72], who reported that an increased androgen-to-estrogen ratio can promote visceral fat accumulation in rodents by inhibiting AMPK activation and stimulating lipogenesis.

Interestingly, after 8 weeks of treatment, the HFHC diet switched to the CTRL diet, to the HFHCco diet or to the HFHCsil diet, and this change resulted in a partial reduction in the negative effects of the obesogenic diet on some steroid hormones. The switch from the HFHC diet to the CTRL was the optimal therapy because the body weight and almost all the hormonal parameters analyzed were close to those observed for the CTRL diet group and in agreement with previous studies [73,74].

In addition to conventional therapies, the use of functional foods and nutraceuticals may represent a novel therapeutic approach to prevent or attenuate diet-related diseases because of their ability to exert anti-inflammatory, antioxidant, and hepatoprotective effects [75,76]. Silymarin [77] and coconut oil [78] are able to influence steroidogenic pathways through the modulation of steroidogenic enzymes, which are altered in many pathological conditions [79]. However, these supplements can support but not replace a balanced standard diet.

As a limitation of the present study, we acknowledge that the exact amount of silymarin that made an effect remains uncertain, and further bioavailability studies are necessary. Furthermore, the absence of a CTRLco group, which prevents a clear assessment of the independent metabolic effects of coconut oil. However, adding coconut oil to the control diet would have transformed it into an HFHC group, thereby potentially confounding the interpretation of the results.

## 5. Conclusions

The switch from HFHC→CTRL diet was the optimal therapy because the body weight and almost all the hormones were close to those observed for the CTRL diet group. Hair concentrations of E2 in adult mice fed the HFHC diet resulted significantly lower than those in CTRL animals, indicating a diet-induced impairment of estrogenic homeostasis in the HFHC group. The inability to reduce obesity in the fat-supplemented group may be related to a lack of aromatase activity, resulting in low estrogen levels accompanied by high DHEA-S concentrations.

However, a nutraceutical supplement in obese subjects may also represent a novel therapeutic approach to prevent or attenuate diet-related disease. Our results demonstrate that supplement of SIL or Coconut oil proves to be a tool capable of modulating endocrine pathways. However, these supplements should be considered as supportive measures rather than substitutes for dietary correction.

## Figures and Tables

**Table 1:** Details of antibodies.

Hormone	Antibody Dilution	Immunized Animal	Antibody Source	Cross-Reactivities (%)	Assay Sensitivity (pg/mL)	Intra-Assay CV (%)	Inter-Assay CV (%)	Standard Curve Equation	r
Cortisol	1:20,000	Rabbit	Biogenesis (Poole, UK) Cat No 2330–5109 Lot 24120652	Cortisol 100%; corticosterone 1.8%; aldosterone < 0.02%	24.6	3.7	10.1	y = 0.97x + 1.15	0.99
DHEA	1:80,000	Rabbit	Analytical Antibodies (Bologna, Italy) Lot RC/14/06	DHEA 100%; 5-androsten-3β,17β-diol 9.2%; epiandrosterone 2.8%; pregnenolone 1.8%; 5α-androstane-3β,17β-diol 0.6%; cholesterol 0.2%; testosterone 0.1%; androstenedione 0.1%; DHEA sulfate 0.04%; cortisol < 0.001%	12.4	3.8	10.6	y = 0.97x + 1.38	0.99
DHEA-S	1:80,000	Rabbit	Analytical Antibodies (Bologna, Italy) Lot RE/96/710	DHEA-S 100%; DHEA glucuronide 13.9%; androstenedione 8.9%; pregnenolone 2.2%; epiandrosterone glucuronide 0.5%; androsterone sulfate 0.4%; cortisone < 0.001%	10.8	3.2	11.8	y = 0.90x + 3.74	0.99
Progesterone (P4)	1:8000	Rabbit	In-house (laboratory) Lot 1	11β-OH-progesterone 46%; 17α-OH-progesterone 0.4%; 20α-OH-progesterone 0.04%; testosterone 0.08%; cortisol < 0.01%; 17β-estradiol < 0.01%; 17α-estradiol < 0.01%; estrone < 0.01%	11.2	3.4	8.2	y = 0.97x + 0.75	0.99
Estradiol (E2)	1:80,000	Rabbit	In-house (laboratory) Lot 1	17β-estradiol 100%; estrone 2.5%; estriol 0.12%; DHEA 0.007%; 17α-estradiol < 0.004%; progesterone < 0.004%; testosterone < 0.004%; androstenedione < 0.004%	15.4	3.7	12.1	y = 0.98x + 1.69	0.99
Testosterone	1:160,000	Rabbit	Analytical Antibodies (Bologna, Italy) Lot RA/86/111	Testosterone 100%; 5α-dihydrotestosterone 43.2%; 5α-androstanedione 33.1%; 5β-androstanedione 11.4%; 5α-androstan-3α,17β-diol 9.4%; androstenedione 0.4%; progesterone 0.01%; DHEA 0.01%; 17β-estradiol 0.01%; cortisol < 0.001%	6.6	4.4	11.5	y = 1.02x − 3.76	0.99

**Table 2:** Estimated marginal means (95% confidence interval: lower-upper–upper) of live weight, hormones and their ratio in hair of mice as affected by diet type at 4 weeks of treatments.

	Diet		*p*-Value
	CTRL	HFHC	
LW (g)	20.9 (20.1–21.7)	23.0 (22.2–23.7)	<0.01
Cortisol (pg/mg of hair)	1.23 (0.92–1.54)	1.66 (1.35–1.97)	0.06
DHEA (pg/mg of hair)	81.30 (68.85–93.75)	60.16 (47.72–72.61)	0.02
Cortisol/DHEA*100	1.52 (1.26–1.78)	2.75 (2.49–3.01)	<0.01
Testosterone (pg/mg of hair)	5.27 (2.53–8.00)	8.56 (5.83–11.3)	0.09
P4 (pg/mg of hair)	16.10 (10.74–21.46)	24.37 (19.0–29.73)	0.04
E2 (pg/mg of hair)	1.23 (1.06–1.40)	1.49 (1.32–1.66)	0.04
DHEA/DHEA-S	1.97 (1.78–2.15)	1.22 (1.03–1.40)	<0.01

CTRL: control diet; HFHC: high-fat high-carbohydrates diet; LW: live weight.

**Table 3:** Estimated marginal means (95% confidence interval: lower-upper–upper) of live weight, hormones and their ratio in hair of mice as affected by diet type at 8 weeks of treatments.

	Diet		*p*-Value
	CTRL	HFHC	
LW (g)	25.3 (19.2–31.5)	32.8 (26.6–38.9)	0.09
Cortisol (pg/mg of hair)	1.39 (1.11–1.67)	1.72 (1.44–2.00)	0.09
Cortisol/DHEA*100	2.10 (1.53–2.67)	2.86 (2.29–3.43)	0.06
P4 (pg/mg of hair)	17.40 (13.5–21.3)	24.03 (20.12–27.94)	0.02
E2 (pg/mg of hair)	1.27 (1.15–1.39)	1.45 (1.32–1.58)	0.05
DHEA/DHEA-S	1.66 (1.50–1.82)	1.35 (1.19–1.51)	0.01

CTRL: control diet; HFHC: high-fat high-carbohydrates diet; LW: live weight.

**Table 4:** Estimated marginal means (95% confidence interval: lower-upper–upper) of live weight, hormones and their ratio in hair mice as affected by diet type at 20 weeks of treatments.

	Diet					
	CTRL-CTRL	HFHC-CTRL	HFHC-CTRLsil	HFHC-HFHC	HFHC-HFHCco	HFHC-HFHCsil
LW (g)	26.29 ^A^ (23.59–29.00)	28.57 ^A^ (25.44–31.69)	27.26 ^A^ (23.71–30.80)	39.42 ^B^ (36.46–42.38)	39.94 ^B^ (37.11–42.76)	38.02 ^B^ (35.51–40.52)
Cortisol (pg/mg of hair)	1.81 ^a^ (1.49–2.12)	1.96 ^ab^ (1.60–2.32)	2.02 ^ab^ (1.61–2.43)	1.99 ^ab^ (1.65–2.33)	1.70 ^a^ (1.38–2.03)	2.32 ^b^ (2.03–2.61)
P4 (pg/mg of hair)	22.89 ^a^ (18.15–27.63)	19.81 ^a^ (14.32–25.29)	28.40 ^ab^ (22.18–34.62)	33.34 ^bc^ (28.14–38.53)	27.32 ^ab^ (22.36–32.27)	40.51 ^c^ (36.11–44.91)
E2 (pg/mg of hair)	25.52 ^c^ (16.63–39.17)	8.04 ^b^ (4.90–13.20)	4.61 ^b^ (2.63–8.09)	1.85 ^a^ (1.16–2.96)	5.33 ^b^ (3.41–8.34)	5.36 ^b^ (3.60–7.98)
Testosterone/E2	0.63 ^a^ (0.24–1.15)	1.51 ^ab^ (0.83–2.44)	3.11 ^b^ (1.88–4.87)	8.16 ^c^ (5.79–11.34)	2.57 ^b^ (1.69–3.75)	2.65 ^b^ (1.84–3.70)
DHEA-S (pg/mg of hair)	45.70 ^A^ (38.38–54.42)	43.67 ^A^ (35.69–53.43)	56.32 ^AB^ (44.80–70.80)	69.83 ^B^ (57.68–84.55)	45.88 ^A^ (38.22–55.06)	56.44 ^AB^ (48.00–66.37)
DHEA/DHEA-S	1.66 ^c^ (1.44–1.87)	1.64 ^c^ (1.39–1.89)	1.31 ^abc^ (1.03–1.59)	1.10 ^a^ (0.86–1.34)	1.57 ^bc^ (1.35–1.80)	1.26 ^ab^ (1.06–1.46)
Cortisol/DHEA-S*100	4.16 ^a^ (3.46–4.86)	4.83 ^a^ (4.02–5.64)	3.74 ^ab^ (2.82–4.65)	2.96 ^b^ (2.19–3.73)	3.83 ^ab^ (3.10–4.56)	4.17 ^a^ (3.53–4.82)

CTRL: control diet; HFHC: high-fat high-carbohydrates diet; CTRLsil: CTRL diet enriched with silymarin; HFHCco: HFHC diet enriched with coconut oil; HFHCsil: HFHC diet enriched with silymarin; LW: live weight. Superscripts letters were assigned based on multiple comparisons of means using Sidak test after a general linear model that considers diet and sex as fixed and blocking factors, respectively (^A,B^: *p* < 0.01; ^a,b,c^: *p* < 0.05).

## Data Availability

The original contributions presented in the study are included in the article, further inquiries can be directed to the corresponding author.
